# Decreased MEF2A Expression Regulated by Its Enhancer Methylation Inhibits Autophagy and May Play an Important Role in the Progression of Alzheimer’s Disease

**DOI:** 10.3389/fnins.2021.682247

**Published:** 2021-06-16

**Authors:** Hui Li, Feng Wang, Xuqi Guo, Yugang Jiang

**Affiliations:** Tianjin Institute of Environmental and Operational Medicine, Tianjin, China

**Keywords:** Alzheimer’s disease, autophagy, MEF2A, microglia, enhancer, methylation

## Abstract

Alzheimer’s disease (AD) is a neurodegenerative disease characterized by amyloid plaques and neurofibrillary tangles which significantly affects people’s life quality. Recently, AD has been found to be closely related to autophagy. The aim of this study was to identify autophagy-related genes associated with the pathogenesis of AD from multiple types of microarray and sequencing datasets using bioinformatics methods and to investigate their role in the pathogenesis of AD in order to identify novel strategies to prevent and treat AD. Our results showed that the autophagy-related genes were significantly downregulated in AD and correlated with the pathological progression. Furthermore, enrichment analysis showed that these autophagy-related genes were regulated by the transcription factor myocyte enhancer factor 2A (MEF2A), which had been confirmed using si-MEF2A. Moreover, the single-cell sequencing data suggested that MEF2A was highly expressed in microglia. Methylation microarray analysis showed that the methylation level of the enhancer region of MEF2A in AD was significantly increased. In conclusion, our results suggest that AD related to the increased methylation level of MEF2A enhancer reduces the expression of MEF2A and downregulates the expression of autophagy-related genes which are closely associated with AD pathogenesis, thereby inhibiting autophagy.

## Introduction

With an increasing aging population, the prevalence of cognitive impairment and neurodegenerative disease has increased. Alzheimer’s disease (AD), the most common form of dementia, is characterized by progressive cognitive impairment and behavioral disorders. The main pathological features of AD are amyloid plaque deposition and neurofibrillary tangles (NFTs), which are neurotoxic and cause neuronal loss, synapse reduction, neurological degeneration, and brain atrophy. Pathological studies have demonstrated that NFTs and amyloid plaque deposition initially occurred in the cortical and hippocampal tissues of AD and subsequently spread to the whole brain (Braak and Braak, 1995). Inhibiting neurotoxicity by reducing amyloid plaque deposition and NFTs has been unsuccessfully attempted (Doody et al., 2013; Ostrowitzki et al., 2017).

Autophagy consists of a series of complex physiological processes in cells, which can eliminate misfolded proteins and damaged organelles, promote the synthesis of biofilms and transport of vesicles, and thus play a key role in reshaping the cell structure and regulating energy metabolism, resisting adverse external stimuli and stabilizing cell homeostasis. Autophagy disorders are closely related to AD (Nixon et al., 2005). Inhibiting lysosomal proteolysis produces similar neuropathological manifestations in wild-type mice and exacerbates amyloid plaque deposition and autophagy pathology in mouse models of AD (Nixon and Yang, 2011). Presenilin 1 mutations, associated with familial AD, result in decreased maturation of the lysosomal v-ATPase and, thus, directly increased lysosomal pH and impaired lysosome function, which would be predicted to reduce autophagosome clearance (Lee et al., 2010). Another genetic risk factor for AD is mutations in apolipoprotein E 4 (ApoE4). ApoE4 destabilizes lysosomal membranes in an allele-specific manner. Other factors include reactive oxygen species and amyloid plaque and oxidized lipids and lipoproteins, which also contribute to AD by impeding lysosomal proteolysis, damaging lysosomal membranes, and disrupting lysosomal integrity, thereby releasing proteases that can mediate neuronal cell death (Boya and Kroemer, 2008; Nixon, 2013). Moreover, a study found that autophagy inducers such as rapamycin in 3xTg-AD mice can effectively reduce the deposition of amyloid plaques in the brain and improve cognitive performance (Majumder et al., 2011). However, the relationships between the pathological progression of AD and autophagy-related genes remain unclear, and the mechanisms have not been elucidated.

Systems biology concepts and methods provide multivariate approaches to holistically analyze the larger interactive network of biological pathways and identify important players in AD onset and progression. In this study, multiple datasets were downloaded, and bioinformatics methods were used to analyze the correlation between autophagy-related genes and pathological progression of AD, assessing the reasons for their differential expression, thereby providing new clues for elucidating the mechanism of AD.

## Materials and Methods

### Data Sources

**FIGURE 1:**
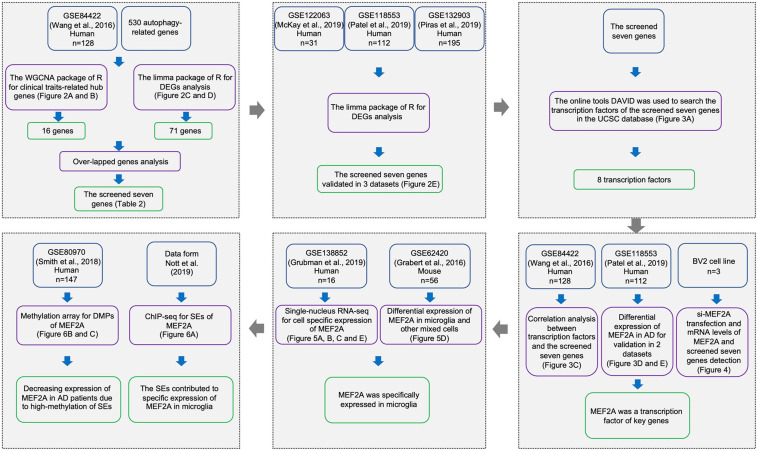
Workflow of sample procedures. The samples and data sources are shown in blue boxes, methods for data processing are shown in purple boxes, and results are shown in green boxes.

Five gene expression profiles of mRNA, one DNA methylation profile, and related clinical data of AD were downloaded from the Gene Expression Omnibus database^1^ (Table 1). The workflow is represented in Figure 1.

**TABLE 1:** Data sources.

**Series ID**	**Platform ID**	**Samples**	**Groups**	**Regions**	**Species**	**Cell types**	**Reference**	**Type**
GSE84422	GPL96/97/570	1,053	Definite AD (328) Healthy controls (214) Possible AD (229) Probable AD (180)	19 cortical regions	*Homo sapiens*	Mixed cells	PMID: 27799057	Expression profiling by array
GSE122063	GPL16699	31	AD (12) Vascular dementia (8) Healthy controls (11)	Frontal and temporal cortex	*Homo sapiens*	Mixed cells	PMID: 30990880	Expression profiling by array
GSE118553	GPL10558	401	AD (167) AsymAD (134) Healthy controls (100)	Entorhinal cortex, temporal cortex, frontal cortex, and cerebellum brain region	*Homo sapiens*	Mixed cells	PMID: 31063847	Expression profiling by array
GSE132903	GPL10558	195	AD (97) Healthy controls (98)	Middle temporal gyrus	*Homo sapiens*	Mixed cells	PMID: 31256118	Expression profiling by array
GSE80970	GPL13534	147	AD (74) Healthy controls (69)	Superior temporal gyrus and prefrontal cortex	*Homo sapiens*	Mixed cells	PMID: 29550519	Methylation profiling by genome tiling array
GSE62420	GPL11180	56	4 months (24) 12 months (16) 22 months (16)	Cerebellum, cortex, hippocampus, and striatum	*Mus musculus*	Microglia	PMID: 26780511	Expression profiling by array
GSE138852	GPL18573	16	AD (8) Healthy controls (8)	Entorhinal cortex	*Homo sapiens*	Mixed cells	PMID: 31768052	Single nuclei RNA sequencing
								

GSE84422 (Wang et al., 2016) was used to perform a weighted correlation network analysis (WGCNA), which consisted of 1,053 postmortem brain samples across 19 brain regions from 125 participants dying of varying severities of dementia and variable AD-neuropathology severities, including clinical dementia rating (CDR), Braak NFT score (Braak), CERAD diagnoses and ratings of pathology (CERAD), average of neuritic plaque counts in five cardinal cortical regions (PLQ_Mn), sum of CERAD semiquantitative rating scores for all cortical regions examined neuropathologically (NPrSum), and sum of semiquantitative NFT density ratings for all cortical regions examined (NTrSum). GSE84422, GSE122063 (McKay et al., 2019), GSE118553 (Patel et al., 2019), and GSE132903 (Piras et al., 2019) were used to screen and validate differentially expressed genes (DEGs). GSE122063 includes frontal and temporal cortical samples from vascular dementia (*n* = 8), AD (*n* = 12), and healthy controls (*n* = 11). GSE118553 includes 401 human brain samples (entorhinal cortex, temporal cortex, frontal cortex, and cerebellum brain region) from 100 healthy controls, 134 asymptomatic AD (AsymAD), and 167 AD participants. Samples of GSE132903 consisted of middle temporal gyrus between AD (*n* = 97) and healthy controls (*n* = 98). GSE62420 (Grabert et al., 2016) was used to validate differentially expressed myocyte enhancer factor 2A (MEF2A).

The mRNA expression levels in GSE84422, GSE118553, GSE132903, and GSE62420 were measured using Illumina HumanHT-12 V4.0 expression beadchip, and the mRNA expression levels in GSE122063 were measured using Agilent-039494 SurePrint G3 Human GE v2 8 × 60K Microarray 039381.

GSE80970 (Smith et al., 2018) contained prefrontal cortex and superior temporal gyrus tissue from 147 participants with varying levels of AD pathology. DNA modifications for these samples were quantified using the Illumina Infinium Human 450K Methylation Array.

Single-nucleus RNA-seq (snRNA-seq), GSE138852 (Grubman et al., 2019), on the entorhinal cortex from control and AD brains of 16 participants, yielding a total of 13,214 high-quality nuclei, were used to check the gene expression distributions across cells in Alzheimer’s disease brains.

In GSE62420, brains of 4-, 12-, and 22-month-old C57Bl/6J mice were collected and dissected into four regions: cerebellum, cortex, hippocampus, and striatum. Microglia were extracted from each region using a magnetic bead-based approach. Total RNA was immediately isolated for purified microglia and stored (−80°C) until performing microarray analysis of purified microglia and regional brain homogenates (*n* = 56).

The autophagy gene list with a total of 530 autophagy-related genes was derived from the Gene Ontology with the term “autophagy” (GO: 0006914) in the Homo sapiens organism.

### Weighted Gene Coexpression Network Analysis

We extracted the autophagy genes to perform WGCNA with expression data retrieved from GSE84422 (Wang et al., 2016) microarray data. The R package “WGCNA” was applied to find clinical trait-related modules and hub genes among them as previously described (Zhang et al., 2014). The adjacency matrix was transformed into topological overlap matrix. According to the topological overlap matrix-based dissimilarity measure, genes were divided into different gene modules. Herein, we set soft-thresholding power as 6 (scale free *R*^2^ = 0.85), cut height as 0.25, and minimal module size as 10 to identify key modules. The module with the highest correlation with clinical traits (age, sex, race, PIM, pH, CDR, Braak, CERAD, PLQ_Mn, NPrSum, and NTrSum) was selected to explore its biological function through gene ontology (GO) analyses and to screen hub genes. Hub genes were defined as those with gene significance > 0.3 and module membership > 0.8.

### Differential Expression Analysis

For the microarray differential expression analyses of GSE84422 (Wang et al., 2016), robust multichip average (RMA) was used for background correction of raw gene expression matrixes, then log_2_ transformation of expression matrixes. The “affy” R package was used for quantile normalization and median polish algorithm summarization. Next, all gene probes were mapped into gene symbols by the affymetrix annotation files. The “limma” (linear models for microarray data) R package was performed for identifying DEGs between definite AD samples and healthy controls, and the results were visualized using the volcano plot and heat map. Cutoff criteria for screening DEGs were *p* < 0.05 and | log2fold change| ≥ 1.3. The screened seven genes were obtained by taking the intersection of the DEGs of GSE84422 and hub genes related to pathological progression of AD from WGCNA. The differential expression analyses were performed in GSE84422, GSE122063 (McKay et al., 2019), GSE118553 (Patel et al., 2019), and GSE132903 (Piras et al., 2019) datasets and log2fold changes in the screened seven genes and significantly differences (AD versus control expression levels) were represented with the corresponding bar plot.

### Transcription Factors Enrichment

To shed further light on the functions of the candidate genes, DAVID online tools (Huang et al., 2009; Wishart et al., 2009) in the UCSC database were used for transcription factor annotations. The motif matrix profile MA0052.4 of MEF2A was downloaded from JASPAR Fornes et al. (2020), and Find Individual Motif Occurrences (FIMO, Grant et al., 2011) of motif-based sequence analysis tools (MEME suite 5.3.3, Bailey et al., 2009) was used to scan sequences of candidate genes for individual matches to the motif of MEF2A. The positions and sequences of the screened seven genes were inquired in UCSC, and promoters were defined as the 2,000-bp window centered on the transcript start site of genes.

### Correlation Analysis

Correlations between each transcription factor and the expression of downstream genes were analyzed (Pearson’s correlation) in GSE84422 (Wang et al., 2016), and result was represented with a heatmap. The correlation between MEF2A and the screened seven genes was validated (Pearson’s correlation) in GSE118553 (Patel et al., 2019), and result was represented with scatter plots. MEF2A mRNA expression levels (signal intensity) in different groups in GSE84422 and GSE118553 were shown, and *p*-values were calculated using GraphPad. In GSE84422, three groups (control, definite AD, possible AD) were classified according to neuropathology category as measured by CERAD (to unify the results, we combined the diagnosis of possible AD and probe AD into possible AD). In GSE118553, participants in the control group were classified as showing no clinical sign of any form of dementia and no neuropathological evidence of neurodegeneration. Participants in the AsymAD group were defined as clinically dementia free at the time of death, but neuropathological assessment at autopsy revealed hallmark AD pathology. Participants in the AD group had both a clinical diagnosis of AD at death and received confirmation of this diagnosis through neuropathological evaluation at autopsy. A one-way analysis of variance (ANOVA) followed by a least significant difference (LSD) test was used for comparison among groups.

### snRNA-Seq Data Analysis

Single-nucleus RNA-seq data were downloaded from the website^2^ as described by Grubman et al. (2019). We then analyzed the expression of MEF2A in different cell types and different groups. The MEF2A mRNA expression levels (logCounts) in different cell types were represented and significant differences (compared with microglia) were calculated using GraphPad. The expression of MEF2A and screened seven genes (logCounts) in different groups were represented using the ggplot2 package in the R software and significant differences (AD versus control expression levels) were calculated using GraphPad. Following the standardization of GSE62420 (Grabert et al., 2016) microarray data, the signal intensity of MEF2A in microglia and other mixed cell types were shown and significant differences (compared with microglia) were calculated using GraphPad.

### Methylation Array Data Analysis

The ChAMP package in R software (Tian et al., 2017) was used to analyze the differentially methylated positions (DMPs), and the screening condition yielded a value of *p* < 0.05. The DMPs range around MEF2A (from the last gene to the next gene) was approximately 99,676,703–100,882,647 bp of chromosome 15, and the coMET package of the R software (Martin et al., 2015) was used to create a Manhattan plot with a threshold value of *p* = 0.05. These DMPs were annotated by UCSC, and the means of CpG methylation levels in different groups were represented in a violin plot in the enhancers.

### Super Enhancers and Chromatin Interaction Analysis

The super enhancer (SE) analyses, as Nott et al. (2019) described, were performed in UCSC. The ATAC-seq, H3K27ac and H3K4me3 ChIP-seq, and PLAC-seq in all types of cells in the 99,950,000–100,200,000-bp section of chromosome 15 were queried, and the original images were downloaded.

### Cell Culture

The mouse microglia cells (BV2), obtained from the Cell Resource Center, Peking Union Medical College (China), were cultured in Dulbecco’s modified Eagle’s medium (DMEM, Gibco, United States) with 10% fetal bovine serum (Gibco, United States) in 5% CO_2_ at 37°C. For *in vitro* transfection (*n* = 3), the target and control siRNA (GenePharma Co., Ltd., China) were transfected into BV2 cells using Lipofectamine 2000 (Invitrogen, United States) according to the manufacturer’s guidelines. Cells were collected 48 h after transfection. The siRNA sequences were listed (Table 2).

**TABLE 2:** The siRNA sequences of MEF2A.

**siRNA**	**Sequences**	
	**Sense (5′-3′)**	**Antisense (5′-3′)**
si-MEF2A-1	GUGGCAGUCUUGGAAUGAATT	UUCAUUCCAAGACUGCCACTT
si-MEF2A-2	CAGCCACGCUACAUAGAAATT	UUUCUAUGUAGCGUGGCUGTT
si-MEF2A-3	GCUCUAAUAAGCUGUUUCATT	UGAAACAGCUUAUUAGAGCTT
Negative control	UUCUCCGAACGUGUCACGUTT	ACGUGACACGUUCGGAGAATT

### Quantitative Real-Time PCR

Total RNA was extracted using TRIzol^®^ Reagent (Life Technologies, Grand Island, NY, United States) and reverse transcribed into cDNA using PrimeScript^TM^ II 1^st^ Strand cDNA Synthesis Kit for qPCR (TaKaRa, Tokyo, Japan) according to the manufacturer’s instructions. Quantitative real-time PCR was performed to detect the gene mRNA levels using 2 × Universal SYBR Green Fast qPCR Mix (ABclonal, Wuhan, China). Primers were synthesized by Sangon (Sangon Biotech Co., Ltd., Shanghai, China). The qPCR conditions were as follows: 95°C for 3 min and 40 cycles of 95°C for 5 s and 60°C for 30 s. Melting curves were tested to assess the accuracy of the PCR analysis. The 2^–Δ^ ^Δ^ ^Ct^ was calculated to analyze the gene expression levels: ΔCt = Ct (target gene) - Ct (β-actin gene), ΔΔCt = ΔCt (treatment) - ΔCt (control). The primer sequences were listed (Table 3).

**TABLE 3:** The primer sequences of MEF2A and the screened seven genes.

**Genes**	**Sequences**	
	**Forward**	**Reverse**
MEF2A	CAGGTGGTGGCAGTCTTGG	TGCTTATCCTTTGGGCATTCAA
BNIP3	TCCTGGGTAGAACTGCACTTC	GCTGGGCATCCAACAGTATTT
CDK5R1-F	CTGTCCCTATCCCCCAGCTAT	GGCAGCACCGAGATGATGG
HERC1-F	TATAACCTGGAACCCTGTGAACC	TCATGTCGCTTGATGCTCTGT
ITPR1-F	CGTTTTGAGTTTGAAGGCGTTT	CATCTTGCGCCAATTCCCG
OPTN-F	TCACAGGTGGCTACAGGTATC	CCGGAGTTGAGTTTGAGCTG
UBQLN2-F	GCCGAGCCCAAAATCATCAAA	ATCTTTCCGGCGAAAATCAGC
USP33-F	GAGGTTTGTTGTCTCATGTGTCC	GTTCATCTCTGGCGAAGAAGG

### Statistical Analysis Software

RStudio (version 3.6.2) was used for data processing, GraphPad (version 8.0) was used for the calculation of significance and means of the differences, and Adobe Illustrator 2020 (version 24.0.1) was used for image processing.

## Results

### WGCNA and Differential Expression Analysis Were Used to Determine Hub Genes Related to AD Clinical Phenotypes

**FIGURE 2:**
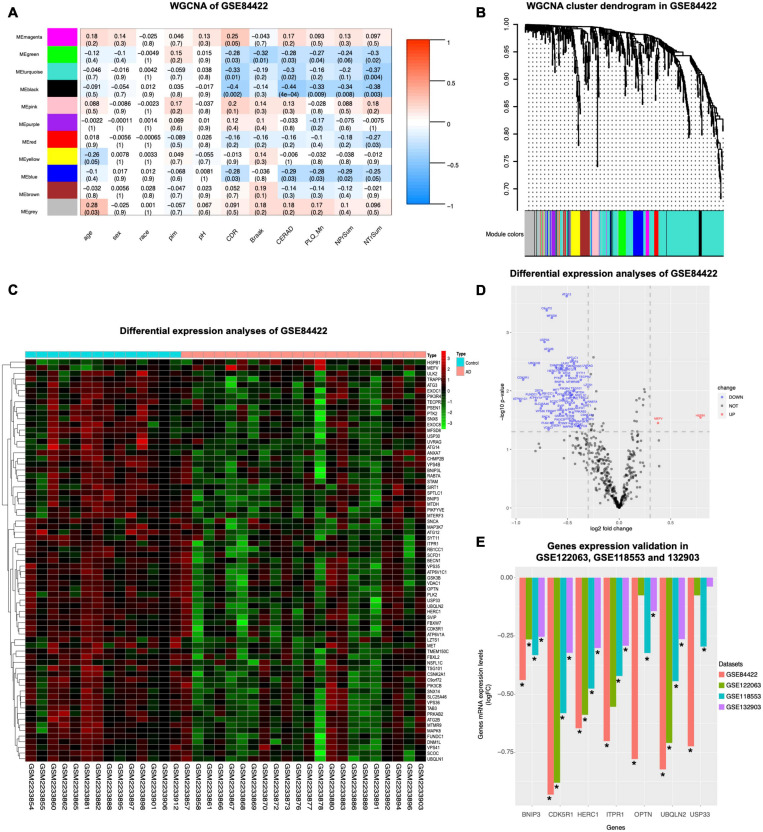
WGCNA coexpression analysis and differential expression analysis. GSE84422 (Wang et al., 2016) was used to perform WGCNA. **(A)** The correlation between each module and clinical phenotypes is shown, including 11 modules and 11 clinical phenotypes. In each unit, the numbers above show the correlation, and the numbers below show the *p*-value. Abbreviations: CDR, clinical dementia rating; Braak, Braak NFT score; CERAD, CERAD diagnoses and ratings of pathology; PLQ_Mn, average of neuritic plaque counts in five cardinal cortical regions; NPrSum, sum of CERAD semiquantitative rating scores for all cortical regions examined neuropathologically; NTrSum, sum of semiquantitative NFT density ratings for all cortical regions examined. **(B)** Cluster dendrogram of the coexpression network modules was produced based on the autophagy genes, including 11 modules. **(C)** DEGs in healthy control and AD in GSE84422 were shown in the heatmap. Cut-off criteria for screening DEGs were *p* < 0.05 and | log2fold change| ≥ 1.3. **(D)** Autophagy genes with significantly different expression in GSE84422 were shown in the volcano plot. Red spots indicate upregulated genes, and blue spots indicate downregulated genes. Cut-off criteria for screening DEGs were *p* < 0.05 and | log2fold change| ≥ 1.3. **(E)** Validation of the gene expression levels of BNIP3, CDK5R1, HERC1, ITPR1, OPTN, UBQLN2, and USP33 between healthy control and AD was shown in three other datasets, GSE122063 (McKay et al., 2019), GSE118553 (Patel et al., 2019), and GSE132903 (Piras et al., 2019). Y-axes in the left indicate log2fold change of the screened seven genes in each dataset. Significant differences (AD versus control expression levels) were performed, *p < 0.05 compared with the control group.

To assess the relationship between gene expression and clinical phenotypes of AD, dataset GSE84422 (Wang et al., 2016) were downloaded and the WGCNA package, which can cluster genes and divide them into different modules and associate the genes of each module with the clinical phenotypes, was used to cluster autophagy genes and divide them into 11 hub gene modules with different module colors, according to the gene expression correlation patterns (Figure 2B). The correlation between each module and clinical phenotypes was calculated (Figure 2A). In the MEblack module, 16 genes included were negatively correlated with CDR, CERAD, PLQ_Mn, NPrSum, and NTrSum (*p* < 0.05), especially, the correlation with the CERAD was the most significant. The MEgreen module was negatively correlated with CDR, Braak, CERAD, PLQ_Mn, and NTrSum (*p* < 0.05); the MEturquoise module was negatively correlated with CDR, CERAD, and NTrSum (*p* < 0.05); the MEred module was negatively correlated with NTrSum (*p* < 0.05); the MEblue module was negatively correlated with CDR, CERAD, PLQ_Mn, and NPrSum (*p* < 0.05); besides, gene expression levels in the MEgrey module were positively correlated with age (*p* < 0.05).

To investigate whether the expression levels of autophagy-related genes were different between healthy controls and AD, we used the limma package to analyze differential expression of autophagy-related genes in definite AD and healthy controls in dataset GSE84422, and then screened 71 DEGs shown in a heatmap (Figure 2C). Compared with healthy controls, the expression of autophagy genes in AD was mostly reduced (69 genes) and only two genes were increased (Figure 2D). The autophagy-related genes of AD were inhibited, suggesting that their autophagy function was lower than that of healthy controls.

Seven genes were screened by taking the intersection of the autophagy-related genes with significant differences and the black module hub genes with strong correlation with pathological progression of AD screened by WGCNA (Table 4). Validating the multiple differences of the screened seven genes (Figure 2E), we found that compared with the control group, all screened seven genes showed a significant decrease in the AD group in GSE118553 (Patel et al., 2019; *p* < 0.05); six genes showed a significant decrease in the AD group in GSE132903 (Piras et al., 2019; *p* < 0.05); four genes showed a significant decrease in the AD group in GSE122063 (McKay et al., 2019; *p* < 0.05).

**TABLE 4:** The expression of autophagy-related genes with significant differences in black module.

**Gene symbol**	**Entrez ID**	**Gene name**	**logFC**	***p*-value**
CDK5R1	8851	Cyclin-dependent kinase 5 regulatory subunit 1	−0.9307557	0.0064305
UBQLN2	29978	Ubiquilin 2	−0.8225799	0.0036374
OPTN	10133	Optineurin	−0.7782243	0.0107843
USP33	23032	Ubiquitin-specific peptidase 33	−0.7235492	0.0014619
ITPR1	3708	Inositol 1,4,5-trisphosphate receptor type 1	−0.7013134	0.0137605
HERC1	8925	HECT and RLD domain containing E3 ubiquitin protein ligase family member 1	−0.6453281	0.0049519
BNIP3	664	BCL2 interacting protein 3	−0.4385456	0.0034525

### MEF2A Expression Is Related to the Screened Seven Genes and AD Neuropathological Category

**FIGURE 3:**
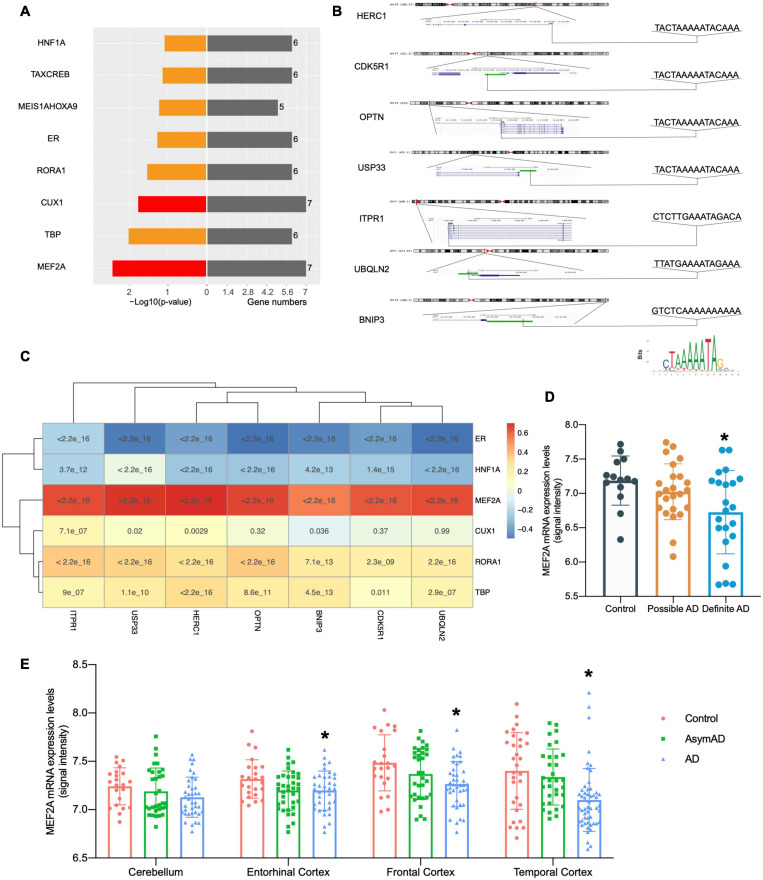
Relationship between MEF2A expression and the screened seven genes. **(A)** Transcription factors were investigated from the UCSC database with online annotation website DAVID; transcription factors correlated with all the screened seven genes are indicated in red. In the left section, -log10(*p*-value) values are shown, and gene numbers enriched are shown in the right section. **(B)** The motifs of the screened seven genes matched to MEF2A (*p* < 0.001) were scanned by MEME tools (described in Methods section “Transcription Factors Enrichment”) and were shown in UCSC. The positions and sequences of the screened seven genes were inquired in UCSC, and promoters (shown as green bar) were defined as the 2,000 bp window centered on the transcript start site of genes. The motif of MEF2A downloaded from JASPAR 2020 is shown on the bottom right. **(C)** Correlations between transcription factors and the expression levels of BNIP3, CDK5R1, HERC1, ITPR1, OPTN, UBQLN2, and USP33 were analyzed (Pearson’s correlation) in the dataset GSE84422 (Wang et al., 2016) and was shown as the heatmap. **(D)** The dataset GSE84422 (Wang et al., 2016) was downloaded, and the mRNA expression (signal intensity) of MEF2A in different neuropathological category of sample (possible AD group, control group, and the definite AD group) were shown using GraphPad. ANOVA followed by LSD test was used for comparison among groups. *p < 0.05 compared with the control group. **(E)** The dataset GSE118553 (Patel et al., 2019) was downloaded and the mRNA expression (signal intensity) of MEF2A in different groups (AD, AsymAD, and control group) and brain regions of sample (cerebellum, entorhinal cortex, the frontal cortex and temporal cortex) were shown using GraphPad. An ANOVA followed by an LSD test was used for comparison among groups. *p < 0.05 compared with the control group.

The online annotation website DAVID was used to perform transcription factor analysis for 71 DEGs and 16 genes in the black module (total 80 genes) in the UCSC database. The result showed that 18 transcription factors were identified by enrichment analysis of 80 genes (*p* < 0.05), including 73 genes enriched in organic cation transporter 1 (OCT1), 66 genes enriched in Ecotropic Virus Integration Site 1 (EVI1), and 65 genes enriched in MEF2 (Supplementary Figure 1). This data suggested that these 18 transcription factors were likely to play important roles in the regulation of autophagy genes in AD. To further investigate the autophagy genes with differences and related to pathological progression of AD, the screened seven genes were annotated by DAVID, revealing that the screened seven genes were regulated by transcription factors MEF2A and CUX1 (Figure 3A). Transcription factors can specifically bind to target motifs to regulate the expression of downstream genes; therefore, the expression of transcription factors was consistent with the expression of downstream genes. We analyzed the correlation between transcription factors and the expression of the screened seven genes obtained from the query of the UCSC database in GSE84422 (Wang et al., 2016; Figure 3C). Among them, the expression of MEF2A was most correlated to the screened seven genes. The motifs of the screened seven genes matched to MEF2A (*p* < 0.001) were scanned by MEME tools (described in Methods section “Transcription Factors Enrichment”) and were shown in UCSC (Figure 3B). The correlation between transcription factors and the expression of the screened seven genes were validated in GSE118553 (Patel et al., 2019; Supplementary Figure 2). These results showed that MEF2A had the strongest correlation with the screened seven genes, so that we selected MEF2A for further study.

We downloaded dataset GSE84422 and extracted the mRNA expression data of MEF2A, and then compared different neuropathological categories of the sample (Figure 3D) and found no significant difference in the possible AD group compared with the control group; in addition, the definite AD group showed a significant difference (*p* < 0.05). The expression of MEF2A in the control group was the highest, followed by the possible AD group, and finally the definite AD group. To validate our results, another dataset GSE118553 was used (Figure 3E). The MEF2A expression levels were significantly lower in the AD group than in the control group (*p* < 0.05) in different brain regions (entorhinal cortex, frontal cortex, and temporal cortex), except for the cerebellum, while AsymAD revealed no significant difference (*p* > 0.05) compared with control.

**FIGURE 4:**
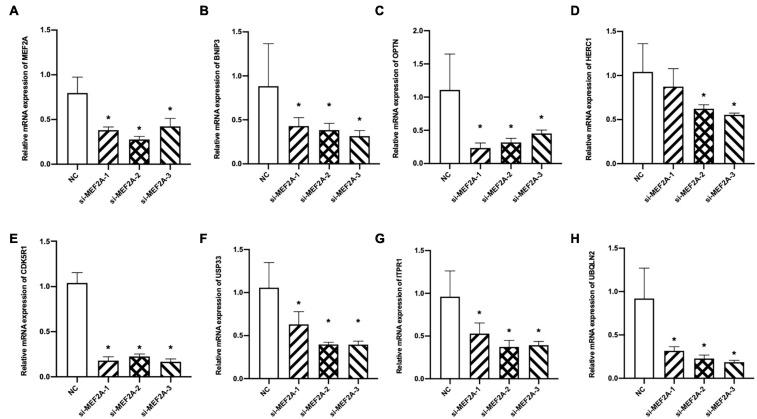
Expression levels of the screened seven genes after si-MEF2A transfection. **(A–H)** After BV2 cells transfected with three si-MEF2A for 48 h to knock down the expression of MEF2A, the expression levels of MEF2A, BNIP3, CDK5R1, HERC1, ITPR1, OPTN, UBQLN2, and USP33 were detected using qPCR. ANOVA followed by LSD test was used for comparison among groups. *p < 0.05 compared with the control group.

In our experiment, to verify the relationship between MEF2A and the screened seven genes, three siRNA were transfected into BV2 cells to knock down the expression of MEF2A, and the mRNA levels of the seven autophagy-related genes were detected using qPCR (Figure 4). These results showed that most of the screened seven genes were significantly decreased following siRNA transfection (*p* < 0.05), suggesting that the expression of the screened seven genes was influenced by MEF2A.

### MEF2A Expression Is Cell Type Specific and Mainly Concentrated in Microglia

**FIGURE 5:**
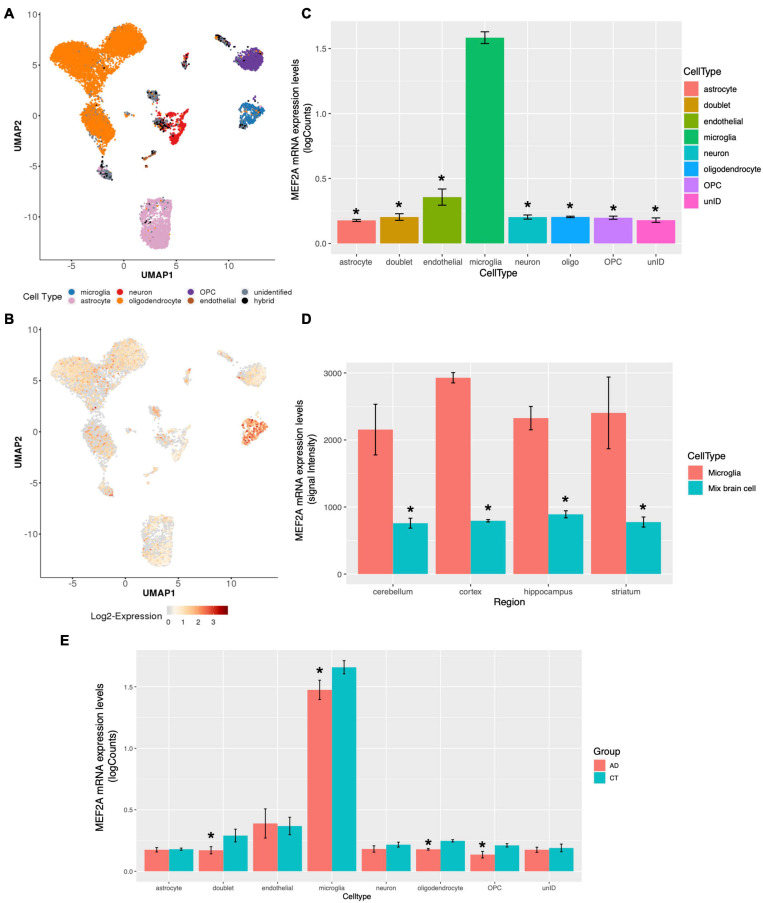
MEF2A expression in brain tissue for different cell types. The snRNA-seq data of GSE138852 (Grubman et al., 2019) were downloaded, and the expression of MEF2A in different cell types were analyzed (A, B, C, E). **(A)** The PCA analysis for cells with different types of markers and cells were clearly divided into eight clusters. **(B)** The differential mRNA expression of MEF2A in each cell types are shown, and the red color indicates high expression. **(C)** The mRNA expression of MEF2A in different cell types was performed using GraphPad. An ANOVA followed by an LSD test were used for comparison among groups. *p < 0.05 compared with the microglia group. **(D)** The mRNA expression of MEF2A in microglia and in mixed brain cell from different brain regions in dataset GSE62420 (Grabert et al., 2016) were performed using GraphPad. An ANOVA followed by an LSD test was used for comparison among groups. *p < 0.05 compared with the microglia group. **(E)** The mRNA expression levels of MEF2A in AD and in healthy controls are shown. *t*-Test was performed for comparison between groups. *p < 0.05 compared with the control group.

The brain contains different cell types, which are responsible for different physiological processes. Therefore, transcriptome sequencing of different subgroups of cells can reflect the functions of different types of cells. Grubman et al. (2019) performed single-cell sequencing on the entorhinal cortex of participants with AD and healthy controls, and the cells with different types of markers were clearly divided into eight subgroups (Figure 5A). The expression levels of MEF2A detected in microglia were significantly higher than those in other subgroups (Figure 5B). The single-cell sequencing dataset, GSE138852 (Grubman et al., 2019), showed that the expression of MEF2A in microglia was significantly (*p* < 0.05) higher than that in other subgroups (Figure 5C), validating the above results. Furthermore, the expression levels of MEF2A in AD were significantly (*p* < 0.05) higher than that in healthy controls in microglia, doublet, oligodendrocyte, and oligodendrocyte progenitor cells (OPC, Figure 5E). Besides, there were no significant difference between AD and healthy controls of the screened seven genes in microglia (*p* > 0.05, Supplementary Figure 3).

In another dataset, GSE62420 (Grabert et al., 2016), microglia from mouse brain tissue were purified according to specific markers, and gene expression was measured. We downloaded the expression matrix and screened the expression levels of MEF2A in each tissue and found that MEF2A was significantly (*p* < 0.05) upregulated in microglia compared with mixed brain cells across different brain regions (Figure 5D). These results suggested that the expression of MEF2A in brain tissue was cell type specific, and the expression of MEF2A in microglia was significantly higher than that in other cells.

### Enhancer Region Methylation Regulates MEF2A Expression

**FIGURE 6:**
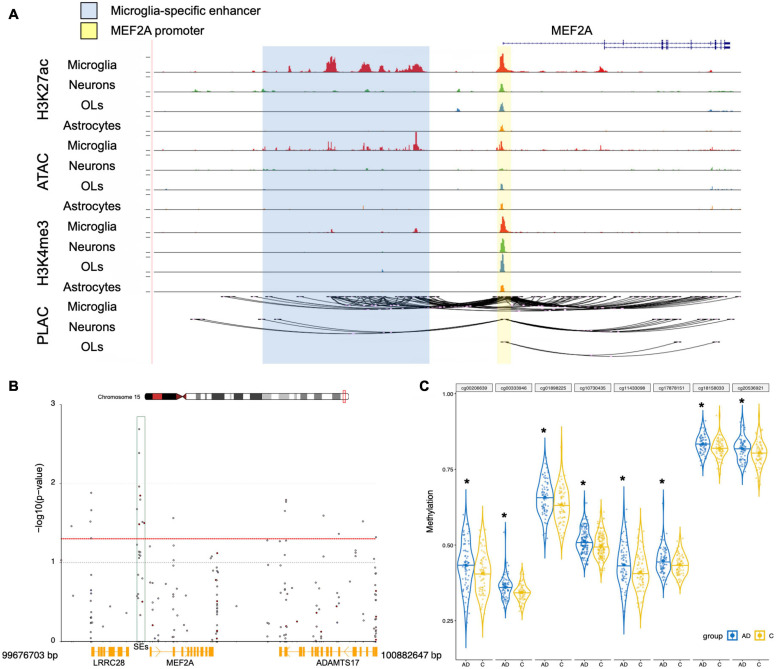
Epigenetic regulation analysis of MEF2A expression. **(A)** UCSC browser of the MEF2A locus showed ATAC-seq, H3K27ac and H3K4me3 ChIP-seq, and PLAC-seq in brain cell types, including neurons, microglia, astrocytes, and oligodendrocytes (OLs). Shared active promoter region is indicated in yellow; microglia-specific enhancer region is indicated in blue. **(B)** The DMPs range around MEF2A, approximately 99,676,703–100,882,647 bp of chromosome 15, was used to create a Manhattan plot by coMET package of the R software with a threshold *p*-value of 0.05 (shown as red dotted line). The -log10(*p*-value) of DMPs for MEF2A in dataset GSE80970 (Smith et al., 2018) were shown in the Manhattan plot. The SEs region is shown as a green box. **(C)** The methylation levels for eight DMPs of the enhancer region in AD and healthy people were shown using GraphPad, and a Student’s *t*-test was used for comparisons across groups. *p < 0.05 compared with the control group.

We then assessed whether epigenetic regulation could alter the expression of MEF2A because both the pathological progression of AD and tissue specificity could change it. SEs are important gene control elements composed of a series of enhancers. We analyzed datasets involved in the interactions between SEs and promoters of different cell types in brain tissue previously published in Nott et al. (2019). We found that ATAC-seq, H3K27ac, and H3K4me3 ChIP-seq showed significant peaks in the MEF2A promoter, with no cell type specificity. The H3K27ac ChIP-seq (a characteristic marker of enhancers and promoters) in microglia showed significant peaks in the MEF2A enhancers, but no significant peaks were observed in neurons or astrocytes. The PLAC-seq revealed a series of regions, mainly concentrated in microglia, which had a high degree of interaction with the promoter of MEF2A, suggesting only microglia had SEs (Figure 6A). Therefore, this suggested that the specific expression of MEF2A in microglia was due to the SEs, which promoted the expression of MEF2A.

We analyzed the DMPs in the dataset GSE80970 (Smith et al., 2018), and the results were shown in a Manhattan plot (Figure 6B). In the SEs region between MEF2A and LRRC28, there were eight DMPs which had methylation differences (*p* < 0.05) in AD compared with healthy controls. The methylation levels of SEs in participants with AD were significantly higher than that in healthy controls (Figure 6C, *p* < 0.05). This suggested that AD may lead to an increase in the methylation of the SE region of MEF2A, thereby reducing the mutual binding with the MEF2A promoter, leading to a decrease in the expression of MEF2A.

## Discussion

In this study, we used bioinformatics methods to investigate the relationship between autophagy-related genes and pathological progression of AD. We downloaded the datasets and identified the seven autophagy-related genes to be correlated with pathological progression of AD using WGCNA. A correlation analysis showed that MEF2A, a transcription factor enriched in UCSC database, was closely related to the screened seven genes. Furthermore, MEF2A was highly expressed in microglia due to the existence of SEs, and the decreased expression of MEF2A in AD was caused by the increased methylation of the SEs.

As a chronic neurodegenerative disease, AD has been found to be closely related to autophagy, given that autophagy-related pathology including accumulated autophagic vacuoles (AVs) were found in a number of dystrophic neurites in AD (Nixon and Yang, 2011). Through live-imaging studies of cortical neurons, this study showed that the inhibition of lysosomal proteolysis could selectively disrupt the axonal transport of autophagy-related compartments, causing an AD-like axonal dystrophy. Our analysis of the array dataset showed that many genes related to autophagy were significantly decreased in AD and closely related to the pathological progression of AD. Among them, gene expression in MEblack module was most strongly associated with CDR, CERAD, PLQ_Mn, NPrSum, and NTrSum, which can reflect the pathology of AD. Thus, genes in MEblack module, including BNIP3, OPTN, CDK5R1, UBQLN2, ITPR1, USP33, and HERC1, were selected for further analysis.

Currently, many studies showed that age-related declines in cognitive fitness were associated with a reduction in autophagy (Hou et al., 2019). However, according to our results, age was positively related to autophagy gene expression in MEgrey module. This may be related to the fact that participants were all over the age of 60. This was consistent with the report of Glatigny et al. (2019) which stated that autophagy was increased in many long-lived model organisms and contributed significantly to their longevity. The relationship between autophagy and longevity warrants further study.

For the genes in MEblack module, the functions of BNIP3, OPTN, and UBQLN2 are related to LC3 on the lysosomal membrane and participate in the fusion process between lysosomes and autophagosomes. BNIP3, a BH3-domain protein of the Bcl-2 family located mainly on the outer membrane of mitochondria, is an important mitochondrial autophagy receptor that can specifically bind to LC3 on the lysosomal membrane to promote the fusion of autophagosomes containing mitochondria with lysosomes to induce autophagy (Chourasia and Macleod, 2015; Tang et al., 2019). OPTN is a ubiquitin-bound autophagy receptor involved in pathogen autophagy and mitochondrial autophagy (Bussi et al., 2018). In aged APP-PSEN1-SREBF2 mice, chronic cholesterol accumulation results in an age-dependent impairment of OPTN translocation to mitochondria, inhibiting mitophagosome formation (Roca-Agujetas et al., 2021). APP/PS1 mice had enhanced Aβ clearance, improved cognition and mobility when treated with miR-331-3p and miR-9-5p, two microRNAs targeting autophagy receptors SQSTM1 and OPTN, respectively, and antagomirs at a late stage (Chen et al., 2021). The function of UBQLN2 in the ubiquitin protease system was to direct misfolded or redundant proteins to the proteasome for degradation. Several studies have shown that UBQLN2 was involved in the process of autophagy and directly combined with LC3 to promote the fusion of lysosomes and autophagosomes (Osaka et al., 2015; Hjerpe et al., 2016; Chen et al., 2018). UBQLN2(P497H) transgenic mice, causes amyotrophic lateral sclerosis (ALS) and frontotemporal type of dementia, had the feature of a dendritic spinopathy with protein aggregation in the dendritic spines and an associated decrease in dendritic spine density and synaptic dysfunction, related to impaired protein degradation (Gorrie et al., 2014).

The other four genes were also closely related to autophagy. CDK5, a serine/threonine kinase, is essential for neuronal migration and synaptic plasticity. CDK5 activation depends on the protein p35 encoded by the CDK5R1 gene, which forms a complex with CDK5 to perform its biological functions (Roufayel and Murshid, 2019). CDK5 genetically interacts with Acinus (Acn), a primarily nuclear protein, which promotes starvation-independent, basal autophagy. Downregulation of CDK5 influences pathologic processes of AD, including the formation of amyloid plaques and tau hyperphosphorylation (Nandi et al., 2017; Nandi and Krämer, 2018). CDK5R1 determines the risk for AD, with a 12.5-fold decrease in AD risk associated with both homozygosity for CDK5R1 (3′-UTR, rs735555) A allele and homozygosity for GSK-3β (-50, rs334558) C allele (Mateo et al., 2009). Moncini et al. (2017) demonstrated that two microRNAs, miR-103 and miR-107, regulate CDK5R1 expression and affect the levels of p35. As the autophagy receptor of cells, ITPR1, a member of the IP3 receptor family, encodes the endoplasmic reticulum (ER) receptor and mediates the release of ER calcium to induce autophagy (Messai et al., 2015; Ren et al., 2017; Xu et al., 2019). Recently, Seo et al. (2020) found ITPR1 displayed associations with the neuroimaging features of AD pathologies through a targeted sequencing analysis of the coding and UTR regions of 132 AD susceptibility genes including 557 participants. USP33 is a deubiquitination enzyme associated with the regulation of lysosomal activity and cell membrane surface receptors (Kommaddi et al., 2015). HERC1, a giant protein belonging to the HERC family, is involved in regulating the ubiquitination of intracellular proteins and can interact with mTOR to regulate the autophagy process (Mashimo et al., 2009; Ruiz et al., 2016; Bachiller et al., 2018).

MEF2A, a member of the MEF2 family, belongs to the MADS-box superfamily and is involved in the transcription of many important genes in the cell life cycle in the form of dimers, including the growth, differentiation and apoptosis of neurons (Zhu et al., 2018). Vargas et al. (2018) used the transcription regulatory network and master regulator analyses on transcriptomic data of human hippocampus from GEO to identify transcription factors that can potentially act as master regulators in AD, and then 34 master regulator candidates were identified including MEF2A. Our results showed that MEF2A, a transcription factor for the seven autophagy-related genes, was significantly decreased in AD. González-Velasco et al. (2020) also reported that 158 genes were regulated by transcription factors MEF2A among the transcriptional changes in the cerebral cortex and hippocampus caused by aging. Genome-wide association studies (GWAS) were performed to assess the significance of the overlap between genome-wide significant AD risk variants and sites of open chromatin from data sets representing diverse tissue types (Tansey et al., 2018). AD risk variants of MEF2A were significantly enriched both in macrophage and microglia. González et al. (2007) stated that variation in the MEF2A gene could be involved in the risk of developing late-onset AD. Besides, MEF2C, another member of the MEF2 transcription factor family, identified by GWAS as also having effects on AD risk, was inferred as a MEF2A target. Moreover, using rapid time-lapse two-photon calcium imaging of network activity and single-neuron growth within the unanesthetized developing brain, Chen et al. (2012) found that MEF2A was the major regulator of neuronal response to plasticity-inducing visual stimulation directing both structural and functional changes.

Microglia, accounting for 10-15% of the total number of brain cells, are an innate immune cell in the central nervous system that uses phagocytosis to engulf apoptotic cells and cellular debris (Plaza-Zabala et al., 2017). Microglia participates in the regulation of tissue repair, synaptic plasticity and synaptogenesis, resist invasion of foreign pathogens, and maintaining the stability of central nervous system tissues. In our study, we found that MEF2A, as a transcription factor to regulate BNIP3, OPTN, and UBQLN2, was highly expressed in microglia. Phagocytosis is very similar to autophagy in vacuole formation and lysosomal digestion. However, unlike autophagy, which is present in all cells, phagocytosis is a unique function of innate immune cells such as microglia. The dysregulation of microglia is closely related to the pathological process of AD, in particular in the context of its role in the phagocytosis of amyloid plaques (Condello et al., 2015; Sacks et al., 2018). The potential regulatory effect of autophagy on phagocytosis may occur in different phases of the phagocytosis cascade, including phagocytosis of substrates, maturation of phagocytes, and fusion with lysosomes, thereby affecting the degradation of phagocytes. For examples, in the LC3-related phagocytosis process, autophagy is partially transferred to phagocytosis to promote the effective intracellular degradation of phagocytic extracellular substances (Martinez et al., 2016). Heckmann et al. (2019) found that the clearance of Aβ in microglia cells correlated with LC3-related phagocytosis, which can promote the phagocytosis efficiency of cells and reuse the membrane receptors related to Aβ phagocytosis, such as CD36, TERM2, and TLR4, to improve cognitive levels.

In the past, microglial phenotypes were characterized by cell surface molecules and were classified as M0, M1 like (exhibiting proinflammatory signaling and neurotoxicity), or M2 like (participating in the resolution of inflammation). However, with the help of newly developed technologies, including single cell RNA sequencing, quantitative proteomics, and epigenetic studies, the characterization of microglial diversity in health and disease has therefore been redefined (Ransohoff, 2016). Recent genome−wide transcriptomic analyses of microglial cells under different disease conditions have uncovered a new subpopulation named disease−associated microglia (MGnD, Verheijen and Sleegers, 2018; García-Revilla et al., 2019). Krasemann et al. (2017) found that MEF2A was significantly decreased in microglia of EAE (multiple sclerosis models), SOD1 (ALS models), and APP/PS1 (AD models) mice. The aggregation of amyloid β (Aβ) changed the M0-homeostatic microglial phenotype to the neurodegenerative phenotype MGnD identified by two major gene clusters, after which the expression of MEF2A was significantly decreased. TREM2 induced APOE signaling and targeting the TREM2-APOE pathway restored the homeostatic signature of microglia in ALS and AD mouse models and prevented neuronal loss in an acute model of neurodegeneration. Our results may explain that the changes in microglia homeostasis in AD were related to autophagy regulated by MEF2A. A genome-wide analysis of gene expression in microglia from different brain regions across the adult lifespan of the mouse was performed, revealing that there were region-specific transcriptional profiles and age-dependent regional variability in gene expression (Grabert et al., 2016). The presence of microglial heterogeneity may underly the different expression patterns of MEF2A in the different brain regions.

The relationship between AD and methylation has been well investigated. Wu et al. (2008) assessed AD-related gene methylation in peripheral blood leucocytes of diagnosed AD, which revealed decreased DNA methylation at the amyloid precursor protein (APP) promoter regions accompanied by upregulated APP transcripts. The methylation of other genes, including BACE1, PSEN1, SORL1, and NEP, involved in amyloidogenic pathway, was also shown to be related to AD (Poon et al., 2020). The epigenetic mechanisms, including the methylation of GSK3β, BDNF, ANK1, BIN1, and RELN, have been consistently reported to play critical roles in neurochemical processes including long-term potentiation (LTP) and synaptic plasticity (Jager et al., 2014; Poon et al., 2020). We found that the MEF2A in AD had significantly higher levels of methylation in the SE region than in healthy controls. SEs are composed of a series of enhancers and are gene control elements with tissue specificity. The significant peaks of H3K27ac ChIP-seq (a characteristic marker of enhancers and promoters) in MEF2A enhancers and promoters in microglia illustrated the reason for which MEF2A is highly expressed in microglia. Changing the methylation levels of SEs can regulate their interaction with gene promoters and thus regulate the expression of related genes (Flam et al., 2019). This suggests that SEs play a key regulatory role in the expression of the MEF2A.

Currently, there is no direct evidence that the screened seven genes are significantly reduced in microglia from the snRNA-seq dataset of GSE138852 (Grubman et al., 2019). However, this does not mean that the screened seven genes are not altered in microglia in AD. We speculate that the expression of these genes may be too low to be accurately detected in microglia, especially OPTN, CDK5R1, and BNIP3, based on the report of Li et al. (2019). Similarly, the low expression levels of these genes do not mean that they are dispensable to microglia. Indeed, these genes are crucial to the function of microglia. Bussi et al. (2018) found that autophagy induced by exogenous fibrillar in microglia correlated with lysosomal damage and was characterized by the recruitment of the selective autophagy-associated proteins TANK-binding kinase 1 (TBK1) and OPTN to ubiquitinated lysosomes. Ma et al. (2013) found that enhanced CDK5 activity by increasing p35-to-p25 conversion promoted Aβ phagocytosis in microglia, whereas the inhibition of CDK5 reduced Aβ internalization. Gong et al. (2020) reported that pinocembrin protected microglial cells against intermittent hypoxia (IH)-induced cytotoxicity by activating BNIP3-dependent mitophagy through the JNK-ERK signaling pathway. In addition, another reason may be linked to the fact that the MGnD was not captured accurately; it was found mainly concentrated around the amyloid plaques and was not evenly distributed throughout AD brain tissue (Krasemann et al., 2017). Besides, whether the reduced expression of these genes in microglia was influenced by epigenetics mechanisms warrants further investigation.

## Conclusion

In summary, our results indicated that AD is associated with the increased methylation levels of MEF2A enhancer, reducing the expression of MEF2A and downregulating the expression of autophagy-related genes which were closely related to AD pathogenesis, thereby inhibiting autophagy. Although further conformational studies are warranted, our findings provide further insights into the role of MEF2A in the prevention and treatment of AD. The association between the reduction of MEF2A expression and autophagy-related genes in AD warrants further investigations.

## Data Availability Statement

The original contributions presented in the study are included in the article/Supplementary Material, further inquiries can be directed to the corresponding author/s.

## Author Contributions

YJ contributed to the perception and finally approved the version to be published. HL participated the whole work, drafted the article, and did the data analysis. FW and XG collected and downloaded the data. All authors contributed to the article and approved the submitted version.

## Conflict of Interest

The authors declare that the research was conducted in the absence of any commercial or financial relationships that could be construed as a potential conflict of interest.

## Footnotes

1 http://www.ncbi.nlm.nih.gov/geo/

2 http://adsn.ddnetbio.com

## Supplementary Material

Supplementary Figure 1 Transcription factor analysis in UCSC database. The online annotation website DAVID was used to performed transcription factor analysis for 71 DEGs and 16 genes in the black module (total 80 genes) in the UCSC database.

Supplementary Figure 2 The correlation between MEF2A and the expression of the screened seven genes. The correlation between MEF2A and the expression of the screened seven genes were validated in GSE118553 (Pearson’s correlation).

Supplementary Figure 3 The mRNA expression levels of the screened seven genes in microglia. In GSE138852, the mRNA expression levels (logCounts) of BNIP3, CDK5R1, HERC1, ITPR1, OPTN, UBQLN2, and USP33 in AD and in healthy controls were shown. A *t*-test was performed for comparison between groups. ^∗^*p* < 0.05 compared with the control group.

## Abbreviations

AD: Alzheimer’s disease
NFT: neurofibrillary tangle
CDR: clinical dementia rating
PLQ_Mn: average of neuritic plaque counts
NPrSum: sum of scores for all cortical regions examined neuropathologically
snRNA-seq: single-nucleus RNA-seq
NTrSum: sum of semiquantitative NFT density ratings for all cortical regions examined
PPI: protein-protein interaction
ANOVA: analysis of variance
LSD: least significant difference
WGCNA: weighted correlation network analysis
SEs: super enhancers
DMPs: differentially methylated positions
DEGs: differentially expressed genes
MEF2A: myocyte enhancer factor 2A.

## References

[B1] Bachiller S. Roca-Ceballos M. A. García-Domínguez I. Pérez-Villegas E. M. Martos-Carmona D. Pérez-Castro M. Á, et al. (2018). HERC1 ubiquitin ligase is required for normal axonal myelination in the peripheral nervous system. *Mol. Neurobiol.* 55 8856–8868. 10.1007/s12035-018-1021-0 29603094

[B2] Bailey T. L. Boden M. Buske F. A. Frith M. Grant C. E. Clementi L. (2009). MEME SUITE: tools for motif discovery and searching. *Nucleic Acids Res.* 37 W202–W208. 10.1093/nar/gkp335 19458158 PMC2703892

[B3] Boya P. Kroemer G. (2008). Lysosomal membrane permeabilization in cell death. *Oncogene* 27 6434–6451. 10.1038/onc.2008.310 18955971

[B4] Braak H. Braak E. (1995). Staging of Alzheimer’s disease-related neurofibrillary changes. *Neurobiol. Aging* 16 271–278. 10.1016/0197-4580(95)00021-6 7566337

[B5] Bussi C. Ramos J. M. P. Arroyo D. S. Gallea J. I. Ronchi P. Kolovou A. (2018). Alpha-synuclein fibrils recruit TBK1 and OPTN to lysosomal damage sites and induce autophagy in microglial cells. *J. Cell Sci.* 131:jcs226241. 10.1242/jcs.226241 30404831 PMC6518333

[B6] Chen M. Hong C. Yue T. Li H. Duan R. Hu W. (2021). Inhibition of miR-331-3p and miR-9-5p ameliorates Alzheimer’s disease by enhancing autophagy. *Theranostics* 11 2395–2409. 10.7150/thno.47408 33500732 PMC7797673

[B7] Chen S. X. Cherry A. Tari P. K. Podgorski K. Kwong Y. K. K. Haas K. (2012). The transcription factor MEF2 directs developmental visually driven functional and structural metaplasticity. *Cell* 151 41–55. 10.1016/j.cell.2012.08.028 23021214

[B8] Chen T. Huang B. Shi X. Gao L. Huang C. (2018). Mutant UBQLN2(P497H) in motor neurons leads to ALS-like phenotypes and defective autophagy in rats. *Acta Neuropathol. Commun.* 6:122. 10.1186/s40478-018-0627-9 30409191 PMC6225656

[B9] Chourasia A. H. Macleod K. F. (2015). Tumor suppressor functions of BNIP3 and mitophagy. *Autophagy* 11 1937–1938. 10.1080/15548627.2015.1085136 26315353 PMC4824596

[B10] Condello C. Yuan P. Schain A. Grutzendler J. (2015). Microglia constitute a barrier that prevents neurotoxic protofibrillar Aβ42 hotspots around plaques. *Nat. Commun.* 6:6176. 10.1038/ncomms7176 25630253 PMC4311408

[B11] Doody R. S. Raman R. Farlow M. Iwatsubo T. Vellas B. Joffe S. (2013). A phase 3 trial of semagacestat for treatment of Alzheimer’s disease. *N. Engl. J. Med.* 369 341–350. 10.1056/NEJMoa1210951 23883379

[B12] Flam E. L. Danilova L. Kelley D. Z. Stavrovskaya E. Guo T. Considine M. (2019). Differentially methylated super-enhancers regulate target gene expression in human cancer. *Sci. Rep.* 9:15034. 10.1038/s41598-019-51018-x 31636280 PMC6803762

[B13] Fornes O. Castro-Mondragon J. A. Khan A. Lee R. V. D. Zhang X. Richmond P. A. (2020). JASPAR 2020: update of the open-access database of transcription factor binding profiles. *Nucleic Acids Res.* 48 D87–D92. 10.1093/nar/gkz1001 31701148 PMC7145627

[B14] García-Revilla J. Alonso-Bellido I. M. Burguillos M. A. Herrera A. J. Espinosa-Oliva A. M. Ruiz R. (2019). Reformulating pro-oxidant microglia in neurodegeneration. *J. Clin. Med.* 8:1719. 10.3390/jcm8101719 31627485 PMC6832973

[B15] Glatigny M. Moriceau S. Rivagorda M. Ramos-Brossier M. Nascimbeni A. C. Lante F. (2019). Autophagy is required for memory formation and reverses age-related memory decline. *Curr. Biol.* 29 435–448.e8. 10.1016/j.cub.2018.12.021 30661803

[B16] Gong L. Wang X. Gu W. Wu X. (2020). Pinocembrin ameliorates intermittent hypoxia-induced neuroinflammation through BNIP_3_-dependent mitophagy in a murine model of sleep apnea. *J. Neuroinflammation.* 17:337. 10.1186/s12974-020-02014-w 33176803 PMC7656728

[B17] González P. Alvarez V. Menéndez M. Lahoz C. H. Martínez C. Corao A. I. (2007). Myocyte enhancing factor-2A in Alzheimer’s disease: genetic analysis and association with MEF2A-polymorphisms. *Neurosci. Lett.* 411 47–51. 10.1016/j.neulet.2006.09.055 17112666

[B18] González-Velasco O. Papy-García D. Douaron G. L. Sánchez-Santos J. M. Rivas J. D. L. (2020). Transcriptomic landscape, gene signatures and regulatory profile of aging in the human brain. *Biochim. Biophys. Acta. Gene Regul. Mech.* 1863 194491–194508. 10.1016/j.bbagrm.2020.194491 32006715

[B19] Gorrie G. H. Fecto F. Radzicki D. Weiss C. Shi Y. Dong H. (2014). Dendritic spinopathy in transgenic mice expressing ALS/dementia-linked mutant UBQLN2. *Proc. Natl. Acad. Sci. U. S. A.* 111 14524–14529. 10.1073/pnas.1405741111 25246588 PMC4209984

[B20] Grabert K. Michoel T. Karavolos M. H. Clohisey S. Baillie J. K. Stevens M. P. (2016). Microglial brain region-dependent diversity and selective regional sensitivities to aging. *Nat. Neurosci.* 19 504–516. 10.1038/nn.4222 26780511 PMC4768346

[B21] Grant C. E. Bailey T. L. Noble W. S. (2011). FIMO: scanning for occurrences of a given motif. *Bioinformatics* 27 1017–1018. 10.1093/bioinformatics/btr064 21330290 PMC3065696

[B22] Grubman A. Chew G. Ouyang J. F. Sun G. Choo X. Y. McLean C. (2019). A single-cell atlas of entorhinal cortex from individuals with Alzheimer’s disease reveals cell-type-specific gene expression regulation. *Nat. Neurosci.* 22 2087–2097. 10.1038/s41593-019-0539-4 31768052

[B23] Heckmann B. L. Teubner B. J. W. Tummers B. Boada-Romero E. Harris L. Yang M. (2019). LC3-associated endocytosis facilitates b-amyloid clearance and mitigates neurodegeneration in murine Alzheimer’s disease. *Cell* 178 536–551.e14. 10.1016/j.cell.2019.05.056 31257024 PMC6689199

[B24] Hjerpe R. Bett J. S. Keuss M. J. Solovyova A. McWilliams T. G. Johnson C. (2016). UBQLN2 mediates autophagy-independent protein aggregate clearance by the proteasome. *Cell* 166 935–949. 10.1016/j.cell.2016.07.001 27477512 PMC5003816

[B25] Hou Y. Dan X. Babbar M. Wei Y. Hasselbalch S. G. Croteau D. L. (2019). Ageing as a risk factor for neurodegenerative disease. *Nat. Rev. Neurol.* 15 565–581. 10.1038/s41582-019-0244-7 31501588

[B26] Huang D. W. Sherman B. T. Lempicki R. A. (2009). Systematic and integrative analysis of large gene lists using DAVID bioinformatics resources. *Nat. Protoc.* 4 44–57. 10.1038/nprot.2008.211 19131956

[B27] Jager P. L. D. Srivastava G. Lunnon K. Burgess J. Schalkwyk L. C. Yu L. (2014). Alzheimer’s disease: early alterations in brain DNA methylation at ANK1, BIN1, RHBDF2 and other loci. *Nat. Neurosci.* 17 1156–1163. 10.1038/nn.3786 25129075 PMC4292795

[B28] Kommaddi R. P. Jean-Charles P. Y. Shenoy S. K. (2015). Phosphorylation of the deubiquitinase USP20 by protein kinase A regulates post-endocytic trafficking of β2 adrenergic receptors to autophagosomes during physiological stress. *J. Biol. Chem.* 290 8888–8903. 10.1074/jbc.M114.630541 25666616 PMC4423680

[B29] Krasemann S. Madore C. Cialic R. Baufeld C. Calcagno N. Fatimy R. E. (2017). The TREM2-APOE pathway drives the transcriptional phenotype of dysfunctional microglia in neurodegenerative diseases. *Immunity* 47 566–581.e9. 10.1016/j.immuni.2017.08.008 28930663 PMC5719893

[B30] Lee J. Yu W. H. Kumar A. Lee S. Mohan P. S. Peterhoff C. M. (2010). Lysosomal proteolysis and autophagy require presenilin 1 and are disrupted by Alzheimer-related PS1 mutations. *Cell* 141 1146–1158. 10.1016/j.cell.2010.05.008 20541250 PMC3647462

[B31] Li Q. Cheng Z. Zhou L. Darmanis S. Neff N. F. Okamoto J. (2019). Developmental heterogeneity of microglia and brain myeloid cells revealed by deep single-cell RNA sequencing. *Neuron* 101 207–223. 10.1016/j.neuron.2018.12.006 30606613 PMC6336504

[B32] Ma Y. Bao J. Zhao X. Shen H. Lv J. Ma S. (2013). Activated cyclin-dependent kinase 5 promotes microglial phagocytosis of fibrillar β-amyloid by up-regulating lipoprotein lipase expression. *Mol. Cell Proteomics* 12 2833–2844. 10.1074/mcp.M112.026864 23816988 PMC3790294

[B33] Majumder S. Richardson A. Strong R. Oddo S. (2011). Inducing autophagy by rapamycin before, but not after, the formation of plaques and tangles ameliorates cognitive deficits. *PLoS One* 6:e25416. 10.1371/journal.pone.0025416 21980451 PMC3182203

[B34] Martin T. C. Yet I. Tsai P. C. Bell J. T. (2015). coMET: visualisation of regional epigenome-wide association scan results and DNA co-methylation patterns. *BMC Bioinformatics* 16:131. 10.1186/s12859-015-0568-2 25928765 PMC4422463

[B35] Martinez J. Cunha L. D. Park S. Yang M. Lu Q. Orchard R. (2016). Noncanonical autophagy inhibits the auto-inflammatory, lupus-like response to dying cells. *Nature* 533 115–119. 10.1038/nature17950 27096368 PMC4860026

[B36] Mashimo T. Hadjebi O. Amair-Pinedo F. Tsurumi T. Langa F. Serikawa T. (2009). Progressive Purkinje cell degeneration in tambaleante mutant mice is a consequence of a missense mutation in HERC1 E3 ubiquitin ligase. *PLoS Genet.* 5:e1000784. 10.1371/journal.pgen.1000784 20041218 PMC2791161

[B37] Mateo I. Vázquez-Higuera J. L. Sánchez-Juan P. Rodríguez-Rodríguez E. Infante J. García-Gorostiaga I. (2009). Epistasis between tau phosphorylation regulating genes (CDK5R1 and GSK-3beta) and Alzheimer’s disease risk. *Acta. Neurol. Scand.* 120 130–133. 10.1111/j.1600-0404.2008.01128.x 19154537

[B38] McKay E. C. Beck J. S. Khoo S. K. Dykema K. J. Cottingham S. L. Winn M. E. (2019). Peri-infarct upregulation of the oxytocin receptor in vascular dementia. *J. Neuropathol. Exp. Neurol.* 78 436–452. 10.1093/jnen/nlz023 30990880 PMC6467199

[B39] Messai Y. Noman M. Z. Janji B. Hasmim M. Escudier B. Chouaib S. (2015). The autophagy sensor ITPR1 protects renal carcinoma cells from NK-mediated killing. *Autophagy* 10.1080/15548627.2015.1017194 [Epub ahead of print] 25714778

[B40] Moncini S. Lunghi M. Valmadre A. Grasso M. Vescovo V. D. Riva P. (2017). The miR-15/107 family of microRNA genes regulates CDK5R1/p35 with implications for Alzheimer’s disease pathogenesis. *Mol. Neurobiol.* 54 4329–4342. 10.1007/s12035-016-0002-4 27343180

[B41] Nandi N. Krämer H. (2018). Cdk5-mediated Acn/Acinus phosphorylation regulates basal autophagy independently of metabolic stress. *Autophagy* 14 1271–1272. 10.1080/15548627.2018.1441472 29782227 PMC6103673

[B42] Nandi N. Tyra L. K. Stenesen D. Krämer H. (2017). Stress-induced Cdk5 activity enhances cytoprotective basal autophagy in Drosophila melanogaster by phosphorylating acinus at serine 437. *Elife* 6:e30760. 10.7554/eLife.30760 29227247 PMC5760206

[B43] Nixon R. A. (2013). The role of autophagy in neurodegenerative disease. *Nat. Med.* 19 983–997. 10.1038/nm.3232 23921753

[B44] Nixon R. A. Wegiel J. Kumar A. Yu W. H. Peterhoff C. Cataldo A. (2005). Extensive involvement of autophagy in Alzheimer disease: an immuno-electron microscopy study. *J. Neuropathol. Exp. Neurol.* 64 113–122. 10.1093/jnen/64.2.113 15751225

[B45] Nixon R. A. Yang D. (2011). Autophagy failure in Alzheimer’s disease–locating the primary defect. *Neurobiol. Dis.* 43 38–45. 10.1016/j.nbd.2011.01.021 21296668 PMC3096679

[B46] Nott A. Holtman I. R. Coufal N. G. Schlachetzki J. C. M. Yu M. Hu R. (2019). Brain cell type–specific enhancer–promoter interactome maps and disease risk association. *Science* 366 1134–1139. 10.1126/science aay0793 31727856 PMC7028213

[B47] Osaka M. Ito D. Yagi T. Nihei Y. Suzuki N. (2015). Evidence of a link between ubiquilin 2 and optineurin in amyotrophic lateral sclerosis. *Hum. Mol. Genet.* 24 1617–1629. 10.1093/hmg/ddu575 25398946

[B48] Ostrowitzki S. Lasser R. A. Dorflinger E. Scheltens P. Barkhof F. Nikolcheva T. (2017). A phase III randomized trial of gantenerumab in prodromal Alzheimer’s disease. *Alzheimers Res. Ther.* 9:95. 10.1186/s13195-017-0318-y 29221491 PMC5723032

[B49] Patel H. Hodges A. K. Curtis C. Lee S. H. Troakes C. Dobson R. J. B. (2019). Transcriptomic analysis of probable asymptomatic and symptomatic alzheimer brains. *Brain Behav. Immun.* 80 644–656. 10.1016/j.bbi.2019.05.009 31063847

[B50] Piras I. S. Krate J. Delvaux E. Nolz J. Mastroeni D. F. Persico A. M. (2019). Transcriptome changes in the Alzheimer’s disease middle temporal gyrus: importance of RNA metabolism and mitochondria-associated membrane genes. *J. Alzheimers Dis.* 70 691–713. 10.3233/JAD-181113 31256118

[B51] Plaza-Zabala A. Sierra-Torre V. Sierra A. (2017). Autophagy and microglia: novel partners in neurodegeneration and aging. *Int. J. Mol. Sci.* 18:598. 10.3390/ijms18030598 28282924 PMC5372614

[B52] Poon C. H. Tse L. S. R. Lim L. W. (2020). DNA methylation in the pathology of Alzheimer’s disease: from gene to cognition. *Ann. N Y Acad. Sci.* 1475 15–33. 10.1111/nyas.14373 32491215

[B53] Ransohoff R. M. (2016). A polarizing question: do M1 and M2 microglia exist? *Nat. Neurosci.* 19 987–991. 10.1038/nn.4338 27459405

[B54] Ren G. Zhou Y. Liang G. Yang B. Yang M. King A. (2017). General anesthetics regulate autophagy via modulating the inositol 1,4,5-trisphosphate receptor: implications for dual effects of cytoprotection and cytotoxicity. *Sci. Rep.* 7:12378. 10.1038/s41598-017-11607-0 28959036 PMC5620053

[B55] Roca-Agujetas V. Barbero-Camps E. Dios C. D. Podlesniy P. Abadin X. Morales A. (2021). Cholesterol alters mitophagy by impairing optineurin recruitment and lysosomal clearance in Alzheimer’s disease. *Mol. Neurodegener.* 16:15. 10.1186/s13024-021-00435-6 33685483 PMC7941983

[B56] Roufayel R. Murshid N. (2019). CDK5: key regulator of apoptosis and cell survival. *Biomedicines* 7:88. 10.3390/biomedicines7040088 31698798 PMC6966452

[B57] Ruiz R. Pérez-Villegas E. M. Bachiller S. Rosa J. L. Armengol J. A. (2016). HERC 1 ubiquitin ligase mutation affects neocortical, CA3 hippocampal and spinal cord projection neurons: an ultrastructural study. *Front. Neuroanat.* 10:42. 10.3389/fnana.2016.00042 27147983 PMC4834294

[B58] Sacks D. Baxter B. Campbell B. C. V. Carpenter J. S. Cognard C. Dippel D. (2018). Multisociety consensus quality improvement revised consensus statement for endovascular therapy of acute ischemic stroke. *Int. J. Stroke* 13 612–632. 10.1177/1747493018778713 29786478

[B59] Seo J. Byun M. S. Yi D. Lee J. H. Jeon S. Y. Shin S. A. (2020). Genetic associations of in vivo pathology influence Alzheimer’s disease susceptibility. *Alzheimers Res. Ther.* 12:156. 10.1186/s13195-020-00722-2 33213512 PMC7678113

[B60] Smith R. G. Hannon E. Jager P. L. D. Chibnik L. Lott S. J. Condliffe D. (2018). Elevated DNA methylation across a 48-kb region spanning the HOXA gene cluster is associated with Alzheimer’s disease neuropathology. *Alzheimers Dement.* 14 1580–1588. 10.1016/j.jalz.2018.01.017 29550519 PMC6438205

[B61] Tang C. Han H. Liu Z. Liu Y. Yin L. Cai J. (2019). Activation of BNIP3-mediated mitophagy protects against renal ischemia–reperfusion injury. *Cell Death Dis.* 10:677. 10.1038/s41419-019-1899-0 31515472 PMC6742651

[B62] Tansey K. E. Cameron D. Hill M. J. (2018). Genetic risk for Alzheimer’s disease is concentrated in specific macrophage and microglial transcriptional networks. *Genome Med.* 10:14. 10.1186/s13073-018-0523-8 29482603 PMC5828245

[B63] Tian Y. Morris T. J. Webster A. P. Yang Z. Beck S. Feber A. (2017). ChAMP: updated methylation analysis pipeline for Illumina BeadChips. *Bioinformatics* 33 3982–3984. 10.1093/bioinformatics/btx513 28961746 PMC5860089

[B64] Vargas D. M. Bastiani M. A. D. Zimmer E. R. Klamt F. (2018). Alzheimer’s disease master regulators analysis: search for potential molecular targets and drug repositioning candidates. *Alzheimers Res. Ther.* 10:59. 10.1186/s13195-018-0394-7 29935546 PMC6015462

[B65] Verheijen J. Sleegers K. (2018). Understanding Alzheimer disease at the interface between genetics and transcriptomics. *Trends Genet.* 34 434–447. 10.1016/j.tig.2018.02.007 29573818

[B66] Wang M. Roussos P. McKenzie A. Zhou X. Kajiwara Y. Brennand K. J. (2016). Integrative network analysis of nineteen brain regions identifies molecular signatures and networks underlying selective regional vulnerability to Alzheimer’s disease. *Genome Med.* 8:104. 10.1186/s13073-016-0355-3 27799057 PMC5088659

[B67] Wishart D. S. Knox C. Guo A. C. Eisner R. Young N. Gautam B. (2009). HMDB: a knowledgebase for the human metabolome. *Nucleic Acids Res.* 37 D603–D610. 10.1093/nar/gkn810 18953024 PMC2686599

[B68] Wu J. Basha M. R. Brock B. Cox D. P. Cardozo-Pelaez F. McPherson C. A. (2008). Alzheimer’s dis- ease (AD)-like pathology in aged monkeys after infantile exposure to environmental metal lead (Pb): evidence for a developmental origin and environmental link for AD. *J. Neurosci.* 28 3–9. 10.1523/jneurosci.4405-07.2008 18171917 PMC2486412

[B69] Xu S. Wang P. Zhang J. Wu H. Sui S. Zhang J. (2019). Ai-lncRNA EGOT enhancing autophagy sensitizes paclitaxel cytotoxicity via upregulation of ITPR1 expression by RNA-RNA and RNA-protein interactions in human cancer. *Mol. Cancer* 18:89. 10.1186/s12943-019-1017-z 30999914 PMC6471868

[B70] Zhang L. Sun Y. Fei M. Tan C. Wu J. Zheng J. (2014). Disruption of chaperone-mediated autophagydependent degradation of MEF2A by oxidative stress-induced lysosome destabilization. *Autophagy* 10 1015–1035. 10.4161/auto.28477 24879151 PMC4091166

[B71] Zhu B. Carmichael R. E. Valois L. S. Wilkinson K. A. Henley J. M. (2018). The transcription factor MEF2A plays a key role in the differentiation/maturation of rat neural stem cells into neurons. *Biochem. Biophys. Res. Commun.* 500 645–649. 10.1016/j.bbrc.2018.04.125 29678571 PMC5956278

