# Supplementary figures and images for: Decreased MEF2A Expression Regulated by Its Enhancer Methylation Inhibits Autophagy and May Play an Important Role in the Progression of Alzheimer’s Disease
Source: Front Neurosci. 2021 Jun 16;15:682247. doi: 10.3389/fnins.2021.682247 (PMC8242211; doi:10.3389/fnins.2021.682247)

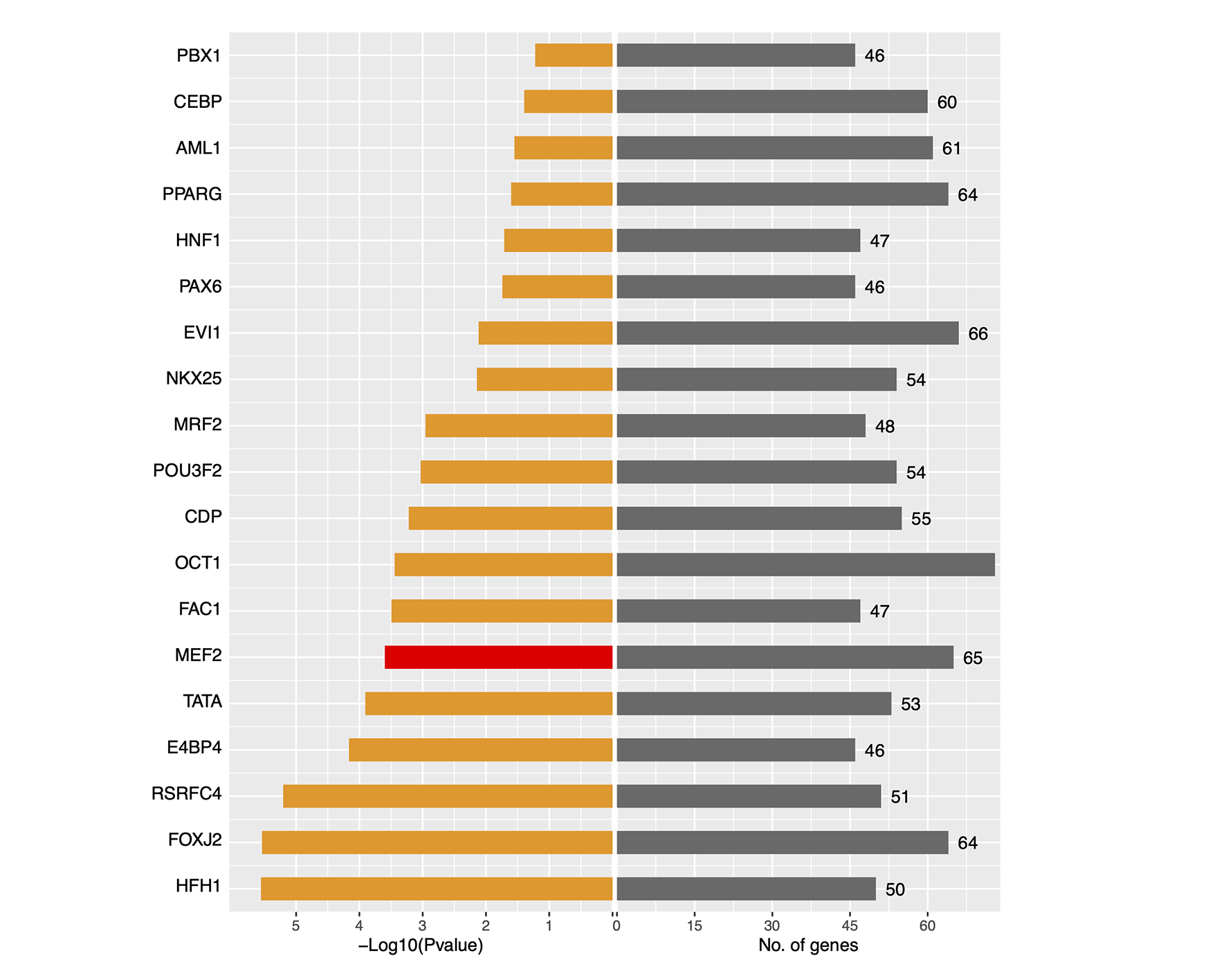

Supplement: Supplementary Figure 1 — Transcription factor analysis in UCSC database. The online annotation website DAVID was used to performed transcription factor analysis for 71 DEGs and 16 genes in the black module (total 80 genes) in the UCSC database. [file Image_1.TIF]

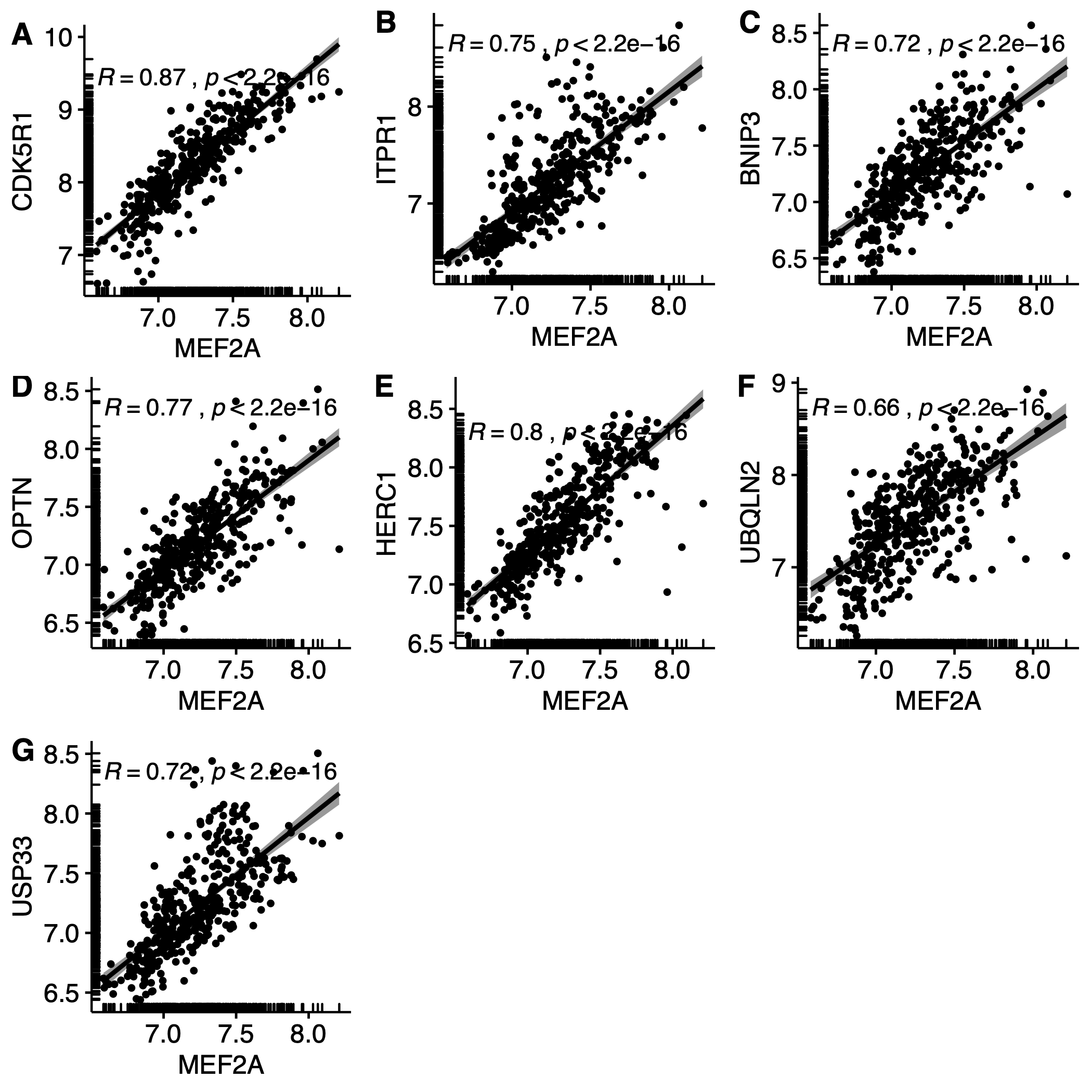

Supplement: Supplementary Figure 2 — The correlation between MEF2A and the expression of the screened seven genes. The correlation between MEF2A and the expression of the screened seven genes were validated in GSE118553 (Pearson’s correlation). [file Image_2.TIF]

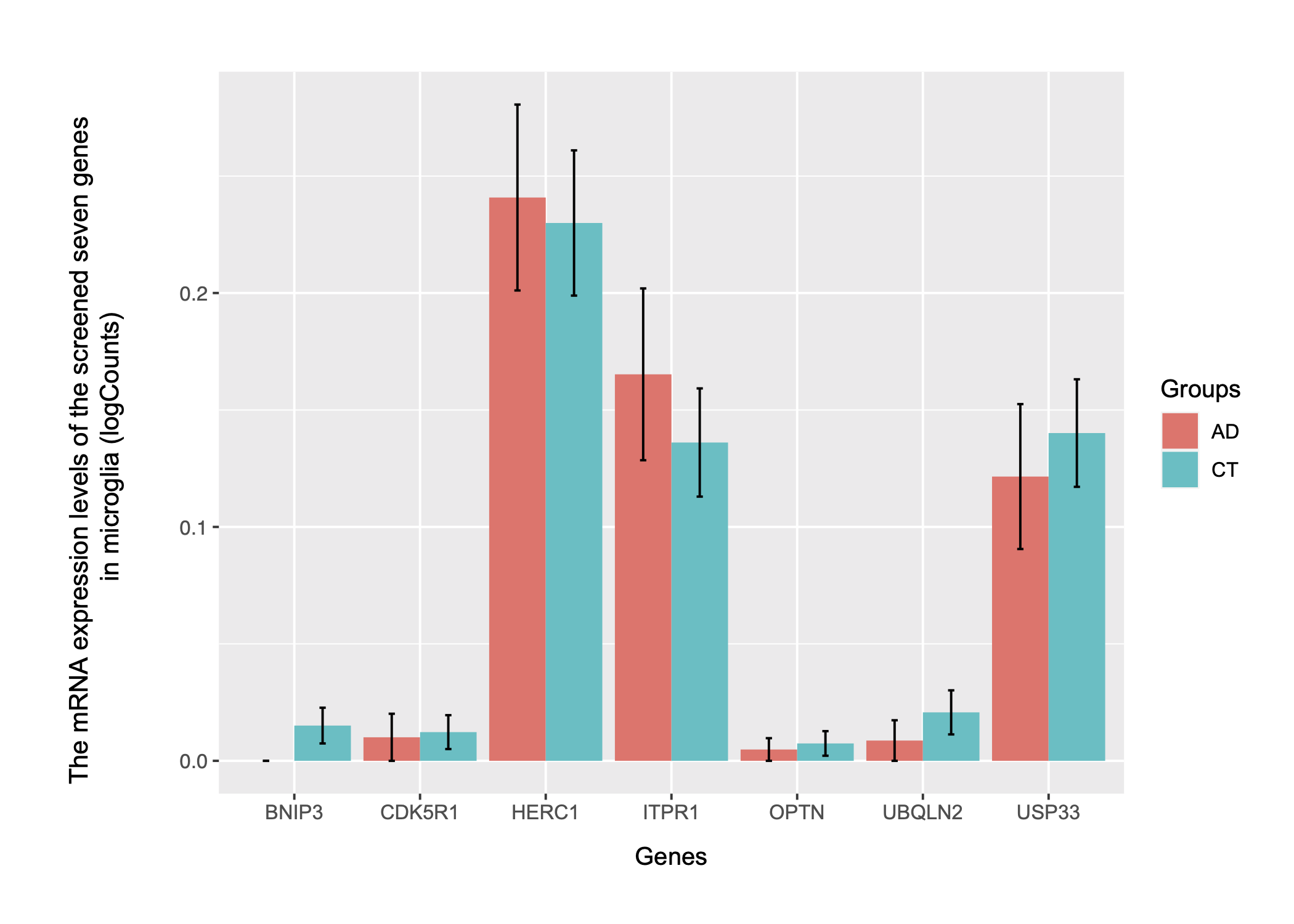

Supplement: Supplementary Figure 3 — The mRNA expression levels of the screened seven genes in microglia. In GSE138852, the mRNA expression levels (logCounts) of BNIP3, CDK5R1, HERC1, ITPR1, OPTN, UBQLN2, and USP33 in AD and in healthy controls were shown. A t-test was performed for comparison between groups. ∗p < 0.05 compared with the control group. [file Image_3.TIF]
